# Wheat sprouts (*Triticum aestivum* Linn.) cultured by a smart farm system ameliorate NAFLD through the AMPK-mediated SREBP signaling pathway

**DOI:** 10.1007/s10068-023-01289-y

**Published:** 2023-04-15

**Authors:** BoYoon Chang, JinHye Bae, SeungBeom Yun, YongDuk Kim, SeongJin Park, SungYeon Kim

**Affiliations:** 1 Institute of Pharmaceutical Research and Development, College of Pharmacy, Wonkwang University, Iksan, 54538 Jeonbuk Republic of Korea; 2 R&D Center, BTC Corporation, #703, Technology Development Center, Gyeongi Technopark, 705, Haean-ro, Sangnok-gu, Ansan-si, 15588 Gyeonggi-do Republic of Korea; 3 Reputer Co., 401, 111-18, Wonjangdong-gil, Deokjin-gu, Jeonju-si, 54810 Jeollabuk-do Republic of Korea

**Keywords:** *Triticum aestivum*, Wheat sprout, Smart farm, WS-S, NAFLD, HFD, AMPK

## Abstract

Wheat is cultivated worldwide and is the most widely distributed food crop. Wheat is a staple crop in many countries. However, the effects of various cultivation methods on the efficacy of wheat sprouts have not been determined. This study investigated wheat sprouts obtained using a standardized smart farm system (WS-S) to improve the effects of non-alcoholic fatty liver disease (NAFLD) and molecular mechanism. Wheat sprouts significantly attenuated the accumulation of lipid droplets in FFA-induced HepG2 cells through AMPK pathway activity. In vivo experiments showed that WS-S significantly lowered body weight gain and decreased adipose tissue, lipid, aspartate transaminase, and alanine aminotransferase levels in HFD/F-treated mice. Furthermore, WS-S stimulated the phosphorylation of ACC and peroxisome proliferator-activated receptor alpha via the AMPK pathway and inhibited SREBP-1/FAS signaling to inhibit de novo adipogenesis and increase fatty acid oxidation. These results suggest that WS-S ameliorates NAFLD by regulating fatty acid metabolism via the AMPK pathway.

## Introduction

Lifestyles are shifting toward healthy living and healthy food, turning to diets rich in fruits and vegetables high in bioactive molecules rather than carbohydrates (Benincasa et al., 2019). Germinated seeds contain 4 to 200 times more vitamins, minerals, and active physiological substances than mature vegetables and are also rich in dietary fiber(Benincasa et al., 2019; 1997). Sprouting is an old practice that improves the nutritional properties of grains (Xiao et al., 2022), increasing the content of simple nutrients after macromolecular degradation. This bioprocess also enhances vitamin and bioactive compound levels. Common seeds used for sprouts include alfalfa (Logan et al., 2001; Zhang et al., 2022), broccoli (Chiriac et al., 2020; Subedi et al., 2019), clover (Dębski et al., 2021; Zhang et al., 2022), radishes (Subedi et al., 2019), rapeseed (Galanty et al., 2022), Chinese cabbage, red cabbage, barley, and wheat (Xiao et al., 2022). However, the contents may differ depending on the cultivation method of the plant (Galanty et al., 2022).

Wheat is an annual grass. Together with rice and corn, it is the world's major food crop. In addition, approximately 30% of the world's population consumes wheat as a staple in bread, crackers, and granola made from the grain itself or grain flour (Logan et al., 2001). In general, wheat sprouts refer to the young shoots of *Triticum aestivum* L. before the formation of nodes during wheat germination.

Mainly young shoots 7–20 days after wheat germination are used. Wheat sprouts are effective against various adult ailments and are often consumed in fresh juice, frozen juice, tablets, and powders. Calcium, potassium, and sodium levels in wheat sprouts have been reported to increase when wheat germinates and grows. Further, wheat sprouts also contain vitamins A, C, and E and essential amino acids, minerals, and enzymes (Aloo et al., 2021). Furthermore, wheatgrass reduces diabetes, inhibits cancer cell growth, exerts anti-inflammatory activity, and has excellent antioxidant and anti-aging properties. Pharmaceutically active ingredients known till date include saponins, flavonoids, alkaloids, terpenes, anthraquinones, anthocyanins, tannins, and glycosides (Moshawih et al., 2022).

Flavonoids from wheat sprouts inhibit adipogenesis in 3T3-L1 cells (Poudel et al.,^ 2015^). In addition, beneficial effects of wheat sprout administration in lipid accumulation animal models, such as obesity (Im et al., 2015; Oh et al., 2019) and diabetes (Adhikary et al., 2021), have been reported.

Germination and plant growth are often affected by various growth factors, including temperature, time, duration, humidity, luminosity, light wavelength, darkness, carbon dioxide, treatment, ozone, and water supply. According to Samuolien et al. (2011), the composition of nutritional and medicinal components of bean sprouts is affected by the intensity, spectrum, and duration of light (Meng et al., 2015). Therefore, methods, such as indoor cultivation or smart farms controlling environmental conditions, are indispensable in sprout production. Therefore, smart farms were established to standardize wheat sprout culture conditions, such as LED light source, ratio, and culture media.

Light is the most important environmental factor affecting photosynthesis and thus yields; plant growth and yield depend on photosynthesis. Supplementary lighting is important for improving plant growth and obtaining year-round yields and quality production. LED sources, such as blue, white, and red, are mainly used for plant cultivation. The growth rate and composition of the components varies depending on the LED light source. In a study by Muthusamy et al., (2020), the contents of saponins and isoorientin in blue and red LED increased from the day of wheat cultivation. Cuong et al. (2019) reported that wheat sprouts grown under blue LED showed slower development than those grown under white and red LED. Jacobsen et al. (2013) found that blue LED light inhibited the germination of grains, restraining the proliferation of barley, brachypodium, and ryegrass. Sun et al. (2017) used a mix of red and white light (2:1) and demonstrated the cell growth acceleration in the low cell density sub-phase for the first time. Since then, research has been conducted on LED mixed lights for plant cultivation (Mao and Guo, 2018; Zhang et al., 2021), for example, to increase the isoorientin content and production in wheat sprouts.

This study investigated the beneficial effects of administering wheat sprouts produced under constant standardized cultivation conditions such as light and nutrients using a smart farm system on the non-alcoholic fatty liver condition. Further, the mechanisms of these effects were examined.

## Materials and methods

### Plant Material and growing conditions

Wheat *(Triticum aestivum* L.) sprouts were provided by BTC Corporation (Sangnok-gu, Ansan, Korea). To set the optimal smart farm cultivation method, the wheat sprouts used in this study were produced by culturing for 11 days using a smart farm system (WS-S) under the grouped blue + water, mix + water, and mix + nutrients. Two lighting regimes (blue and Light mix) were applied. Light mix consisted of white (20%), blue (27%), and red (53%) using LED. During cultivation, plants were supplied with water or nutrient solutions (DAEYU, Kangnam, Korea).

### Growth characteristics

After 11 days of cultivate, plants were harvested, and the growth characteristics were measured fresh weights of shoot. Wheat sprouts were extracted with 50% ethanol for 2 h at 50 °C. The extract was filtered (Whatman No. 4, Whatman plc, Maidstone, UK) and concentrated under reduced pressure at 50 °C (15–30 Brix). Then, it was sterilized at 90 °C for 1 h, freeze-dried, and stored at 4 °C until the experiment. Isoorientin was used as a marker of wheat sprouts for validation.

### High-performance liquid chromatography (HPLC) analysis

**Fig. 1:**
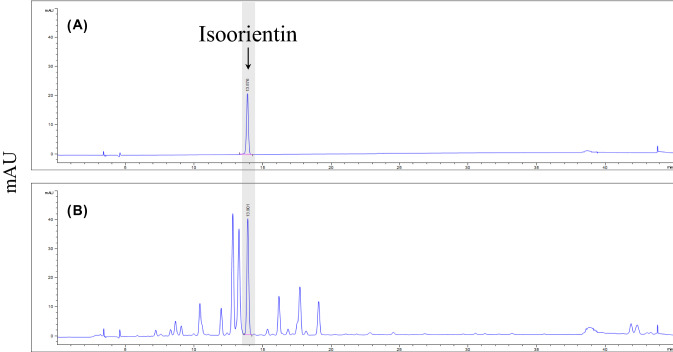
HPLC–DAD chromatograms of **a** isoorientin standard and **b** wheat sprout with excitation at 360 nm

The isoorientin content of the wheat sprouts were determined by HPLC using an Agilent Infinity 1260 series system with a diode array detector (DAD) (Agilent Technologies, Palo Alto, Cam USA). Their separation was conducted at 25 ℃ using a YMC Triart C18 Column (4.6 X 250 mm, 5 μm; YMC KOREA Co., Ltd. Seongnam, Korea). The mobile phase was composed of 0.1% formic acid in distilled water (A) and 0.1% formic acid in acetonitrile (B). The gradient dilution conditions were as follows: 0–20 min, 15–25% B; 20–21 min, 25–100% B; 21–26 min, 100–100% B; 26–27 min, 100–15% B; 27–32 min, 15–15% B. The flow rate was 0.8 mL/min and the injection volume was 3 μL. Chromatograms were obtained at a wavelength of 360 nm (Fig. 1).

### Cell culture and lipid accumulation

HepG2 cells were purchased from the American Type Culture Collection (ATCC, Manassas, VA, USA) and maintained in Dulbecco’s modified eagle medium (DMEM), 1% antibiotics, and 10% fetal bovine serum in a humidified 5% CO_2_ atmosphere at 37 °C. The cells used had fewer than five passages. HepG2 cells were incubated for 24 h in DMEM containing 0.25 mM of a mixture of free fatty acids (FFA; oleic acid: palmitic acid = 2:1) and 1% FFA-free bovine serum albumin to induce intracellular lipid accumulation. HepG2 cells were treated with different concentrations of WS-S and silymarin (100 μM, used as a positive control), 2 h prior to the addition of the FFA mixture.

### Experimental animals and design

Five-week-old C57BL/6 male mice were obtained from Gbio Korea, Inc. (Charles River, Gwangju, Korea). The animals were housed under optimal conditions of humidity (50–55%) and temperature (22–25 °C) with a 12 h light/dark cycle and free access to food and water. The study was approved by the Wonkwang University Animal Care Committee (WK21-35). Mice were divided into five groups (n = 8 per group). The experimental diets used to induce non-alcoholic fatty liver disease (NAFLD) were a 60 kcal% fat diet and 400 kcal% fructose (HFD/F) diet (Rodent Diet D12091402; Research Diets, New Brunswick, NJ, USA). Mice in the normal group were fed a normal diet (ND; Rodent Diet D12450B; Research Diets). The mice were divided into five groups: HFD/F (HFD/F-CON), HFD/F + WS-S (100, 200, and 300 mg/kg), and HFD/F + silymarin (100 mg/kg) groups. The HFD/F received an identical volume of the vehicle.

During the test period, body weight and food intake were measured weekly, and the weight gain and food efficiency were calculated. Mice were anesthetized, and the amounts of fat and muscle in their bodies were measured and analyzed using pDEXA (InAlyzer DXA, Medikors, Seongnam, Korea) three times at 4-week intervals. After all experiments were completed, whole blood was collected and the serum was separated. The wet weights of the harvested organs (liver, spleen, and adipose tissue) were measured. All samples were stored at -80 °C until use.

### Biochemical assays

The liver function was evaluated by measuring ALT and AST serum levels using Frankle's method. In addition, serum triglyceride (TG) (Biovision, CA, USA) and total cholesterol (Asan Co.) (Asan Co., Seoul, Korea) concentrations were quantified colorimetrically using enzymatic kits.

### Histopathology

Hepatic tissues were fixed in 10% buffered neutral formalin solution for 24 h and dehydrated with sucrose to prepare frozen blocks. Frozen sections were cut into 5-μm-thick slices, pasted on glass slides, and stained with Oil Red O and hematoxylin. A microscope at a magnification of 200 × was used to observe lipid accumulation in the liver tissue.

### Gene expression analysis

RNA was separated from each hepatic tissue sample using Easy Blue (iNtRON Biotechnology, Seongnam, Korea). TaqMan one-step master mix (Applied Biosystems, Waltham, MA, USA) was used for reverse transcriptase and real-time PCR of the mRNA samples. PCR was performed using an ABI 7500 Real-Time PCR system (Applied Biosystems, Foster City, CA, USA). Fatty acid synthase (FAS) (Mm01204974_m1), SREBP1 (Mm00550338_m1), tumor necrosis factor (TNF) (Mm00443258_m1), interleukin-6 (IL-6) (Mm00446190_m1), and β-actin (Mm99999915_g1) levels were validated using TaqMan gene expression assays. Relative gene expression was calculated using StepOne software v2.3 (Applied Biosystems, Foster City, CA, USA) through relative threshold cycle (CT) values. β-actin was used as the reference value. Three repetitions were performed for the harvested cell sample and the hepatic sample of animals.

### Western blotting

PROPREP™ Protein Extraction Solution (iNtRON Biotech, Seongnam, Korea) was used with dephosphorylation inhibitors to isolate proteins in cells or liver tissue. Proteins (20 μg each) separated by 10% Sodium dodecyl sulphate–polyacrylamide gel electrophoresis were electroblotted on a polyvinylidene fluoride membrane blocked with 5% skim milk. Primary antibodies (purchased from Cell Signaling Technology (Danvers, MA, USA), AMP-activated protein kinase (AMPK), sterol regulatory element-binding protein 1c (SREBP1c), anti-phospho-ACC, anti-FAS, and anti-peroxisome proliferator-activated receptor alpha (PPARα))were used at 1:1000 dilution. Proteins were detected by the enhanced chemiluminescence method. In addition, the intensities of the protein blots were analyzed using the FluorChem E imaging system (ProteinSimple; Santa Clara, CA, USA). Harvested cell samples and animal hepatic samples were analyzed and evaluated in triplicates.

### Oil Red O staining of intracellular fat

The FFA-induced cells were washed with phosphate-buffered saline (PBS) and fixed with 4% paraformaldehyde for 1 h. After washing with 60% isopropanol, cells were incubated with Oil Red O for 20 min. The staining solution was washed with PBS, and the cells were observed under a microscope. Oil Red O staining of the cells was dissolved with 100% isopropanol, and the lipid content was measured at 540 nm.

### Statistical analysis

Data are expressed as the mean ± S.D. Significant differences were determined using repeated measures ANOVA followed by the Newman-Keuls multiple range test. Statistical significance was defined as *p* < 0.05. All statistical analyses were performed using GraphPad Prism 5.0 (GraphPad Software Inc., San Diego, Ca, USA).

## Results and discussion

### Standardization of smart farm cultivation conditions

**Fig. 2:**
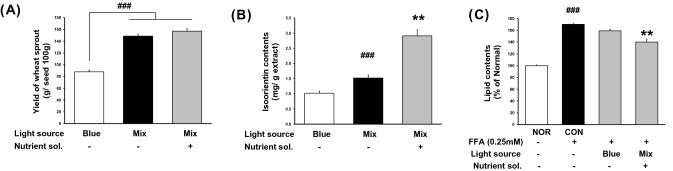
Effect of wheat sprout on culture conditions of the smart farm system. **a** Yield of wheat sprout, **b** isoorientin contents and **c** dissolved cellular lipid contents according to LED source, LED ratio (mixed ratio), and nutrient. Data are presented as the mean ± SD, n = 3. ###*p* < 0.05 compared only Blue or NOR. **p < 0.01 compared to mixed or CON

The shoot length of wheat sprouts was measured 1–11 days after sowing. The length and yield of wheat sprouts improved with the type of LED light and the light ratio. Isoorientin is a natural flavone of interest because of its various pharmacological activities. Plants rich in isoorientin have strong ameliorative properties against complications, such as hyperglycemia (Ziqubu et al., 2020) or hepatic damage (Li et al., 2021). Therefore, the LED lighting conditions were set according to the isoorientin content of the cultivated wheat sprout extract. The content of the isoorientin marker compound also increased in the light mix group (1.52 ± 0.1 mg/g extract) compared to that in the only blue light group (1.02 ± 0.07 mg/g extract). The wheat sprout smart farm's cultivation conditions (nutrient solution, temperature, and humidity) were standardized based on the LED light source and ratio. The yield of grown wheat sprouts was 157.0 ± 0.1 g, and the isoorientin content was 2.91 ± 0.2 mg/g extract in the smart farm system standardized culture method (Fig. 2a, b). We named the wheat sprouts cultivated in the smart farm with a standardized cultivation method as WS-S. We confirmed that WS-S significantly increased the fat accumulation inhibitory effect at the same concentration(100 μg/mL) compared to the blue light group (Fig. 2c).

### Effects of WS-S and isoorientin on anti-adipogenic activity in FFA-stimulated HepG2 cells

**Fig. 3:**
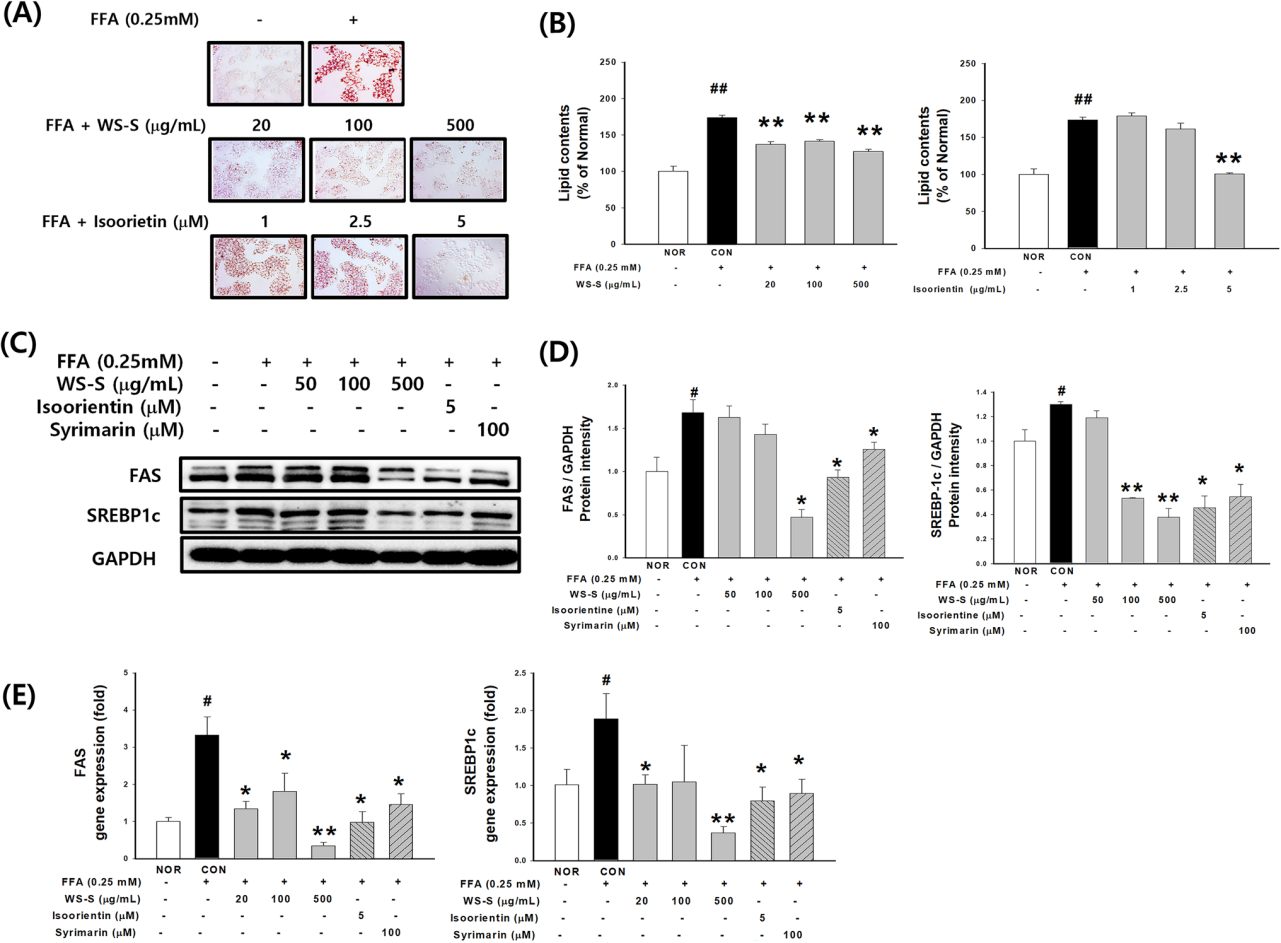
Effect of WS-S and isoorientin on lipid accumulation and lipogenesis in HepG2 cells. **a** Oil Red O (ORO) staining. Dissolved cellular lipid contents of **b** WS-S and isoorientin at 540 nm. **c**, **d** protein expression of SREBP-1 and FAS was determined by western blot; **e** relative mRNA levels of SREBP-1 and FAS were assayed by real-time PCR. Data are presented as the mean ± SD, n = 8. #*p* < 0.05 compared to Normal (NOR). *p < 0.05, **p < 0.01 compared to FFA-induced Control (CON)

At the experimental concentration of 100 μg/mL and 5 μM, cytotoxicity by WS-S and isoorientin treatments was not observed (data not shown). The results (Fig. 3a, b) show an obvious increase in lipid droplets after FFA-induced CON compared to that in NOR. In contrast, the WS-S treatment group showed inhibited lipid accumulation at all concentrations, the 5 μM isoorientin also showed inhibited lipid accumulation in a concentration-dependent manner compared to the CON group. Significant decreases in FAS and SREBP-1c protein and mRNA levels were observed after WS-S and isoorientin treatment (Fig. 3c, d, e). WS-S showed a similar or higher improving effect on lipid accumulation than silymarin, which was used as a positive control.

Ziqubu et al. showed that isoorientin reduces lipid accumulation in mature 3T3-L1 adipocytes. The expression levels of AKT and AMPK proteins were enhanced by isoorientin exposure, suggesting their partial role in modulating lipid metabolism and mitochondrial biogenesis (Ziqubu et al., 2020). Flavonoids purified from *Triticum aestivum* sprouts also significantly inhibit the transcriptional regulators of adipogenesis in 3T3L-1 (Poudel et al., 2015). The expressions of both SREBPs and their target molecules were significantly suppressed or enhanced by WS-S and isoorientin. These data could result from alterations in the synthesis and/or uptake of fatty acids. It is well documented that AMPK phosphorylation inhibits SREBP-1 through the mammalian target of rapamycin (mTOR) and liver X receptor-α (LXRα).

### Effects of WS-S on body composition in NAFLD-induced mice

Food and energy efficiencies (FER and EER) were not significantly higher in the wheat sprout 200 mg/kg group than those in the HFD/F-CON group (data not shown). The HFD/F-CON group consuming the high-fat and high-fructose diet showed a significant difference in body weight in the first week compared to the NOR group consuming the regular diet. At week 12, the WS-S 200 mg/kg group (body weight: 32.97 ± 3.27 g) showed a 6.22% decrease in body weight compared to the HFD/F-CON group (body weight: 35.16 ± 2.99 g). In the wheat sprout 200 mg/kg group, the gain weight was 10.97 ± 3.78 g, a decrease of 14.8% compared to the HFD/F-CON group (12.88 ± 3.50 g).

In the 200 mg/kg WS-S group, fat decreased by 23.37% and lean mass increased by 8.94% compared to the HFD/F-CON group at the 12th week determined by the pDEXA analyzer. The wet weight of abdominal and epididymal fat decreased by 13.9% and 22.76%, respectively. Im et al. (2015) investigated the anti-obesity effects of a 50% EtOH extract of *Triticum aestivum* sprouts in high-fat diet (HFD)-induced obese mice at 6 weeks. *Triticum aestivum* sprouts markedly reduced body weight gain, white fat, and hepatic lipid accumulation. Oh et al. (2019) reported that in the HFD-fed mice at 12 weeks, *Triticum aestivum* extract improved blood lipid profiles, liver inflammation scores, steatosis scores, and obesity compared to vehicle. These findings suggest that WS-S may be effective in treating NAFLD. However, it is not clear what are the major WS-S bioactive compounds and their effect on lipid metabolic enzymes, including fatty acid synthase, sterol regulatory element-binding protein-1c, and the key transcription factors in NAFLD.

Pharmacological studies and new therapeutic strategies for NAFLD can be derived from natural products or materials derived from natural products (Shoreibah et al., 2016; Zobeiri et al., 2018). Previous studies have shown that several Chinese herbal extracts (curcumin (Jazayeri-Tehrani et al., 2019) and resveratrol (Charytoniuk et al., 2017)) prevent the progression of NAFLD in vivo and in vitro. Various animal models have been used to study NAFLD (Recena Aydos et al., 2019). The most widely used mouse model is the induction of NAFLD by a HFD in C57BL/6 mice. A HFD can cause obesity, hyperinsulinemia, hyperglycemia, hypertension, and liver damage in animals, resembling the phenotype observed in humans with NAFLD (Leyva-Jiménez et al., 2020). Therefore, we used this model to induce NAFLD. HFD intake induces obesity and steatosis but does not easily progress to liver inflammation and fibrosis in mice. We fed mice HFD + fructose for 12 weeks to overcome this problem. We investigated the improvements in lipid-accumulated hepatocytes and NAFLD models using WS-S.

Im et al. reported that body weight was reduced by the administration of *Triticum aestivum* sprouts. However, in the present study, WS-S did not cause significant weight loss compared to the HFD/F group. However, fat distribution was significantly decreased when analyzed by the pDXA-body composition. In addition, the white fat (abdominal and epididymal) weight decreased slightly (Im et al., 2015).

### Effects of WS-S on serum biochemistry and lipid contents in NAFLD-induced mice

Alcoholic liver disease, chronic hepatitis B, chronic hepatitis C, alcoholic liver disease, drug-related liver injury, fatty liver, steatohepatitis, and autoimmune hepatitis are common causes of elevated transaminase levels in clinical practice (Jazayeri-Tehrani et al., 2019). The essential step in diagnosing elevated serum aminotransferases is thorough medical history. The alanine aminotransferase (ALT) and aspartate transaminase (AST) blood levels were measured as biochemical indicators of hepatotoxicity. In the WS-S group, the concentration of ALT in the blood at 300 mg/kg WS-S was significantly decreased by 10.04% compared to the HFD/F-CON group. Serum AST concentration also showed a significant decrease of > 38.96% at all doses of wheat sprout. In the WS-S 100 and 300 mg/kg groups, serum TG levels were significantly decreased by 33.2% and 36.05%, respectively, compared to the HFD/F-CON group. In the WS-S 200 mg/kg group, total cholesterol decreased by 13.77% compared to the HFD/F-CON group (Table 1). These results suggest an improved effect of HFD on liver damage.

**Table 1:** Effects of WS-S on serum biochemistry and lipid contents in NAFLD-induced mice

Groups	ALT (unit/mL)	AST (unit/mL)	Triglyceride (mg/dL)	Total cholesterol (mg/dL)
HFD/F	65.4 ± 6.45	88.56 ± 13.05	80.70 ± 31.6	193.78 ± 27.01
HFD/F + WS-S 100 mg/kg	58.33 ± 6.24	68.11 ± 17.04*	51.21 ± 18.33*	198.48 ± 36.71
HFD/F + WS-S 200 mg/kg	63.84 ± 7.55	67.07 ± 11.11*	60.87 ± 18.25	167.09 ± 10.30**
HFD/F + WS-S 300 mg/kg	58.85 ± 5.61*	54.05 ± 6.10*	51.60 ± 12.89*	184.19 ± 22.20
HFD/F + Silymarin	66.47 ± 5.86	64.02 ± 6.15*	77.66 ± 18.36	209.01 ± 30.00

Data are presented as the mean ± SD n = 8

*p < 0.05 and **p < 0.01 compared to the HFD/F group

Increased oxidative stress and activation of inflammatory signaling processes caused by excess FFA directly lead to liver injury, resulting in the accumulation in the liver as a protective mechanism (Charytoniuk et al., 2017). This leads to liver cell death and inadequate repair, ultimately resulting in liver fibrosis and disease progression. In patients with fatty liver, owing to the continuous excess energy supply and the disorder of fatty acid metabolism, fat gradually accumulates in the liver in the form of TG. As a result, the concentrations of TG and FFA in the blood also increase. An increase in hepatic fat is a characteristic finding in the development of NAFLD and the progression of non-alcoholic fatty liver disease. However, qualitative fat composition changes have recently been considered important (Leyva-Jiménez et al., 2020). Histopathological observations suggested that WS-S decreased liver fat in a concentration-dependent manner. Serum TG and TC levels decreased compared with those in the HFD/F group, and serum TC levels were significantly different.

### Effects of WS-S on hepatic inflammation in NAFLD-induced mice

**Fig.4:**
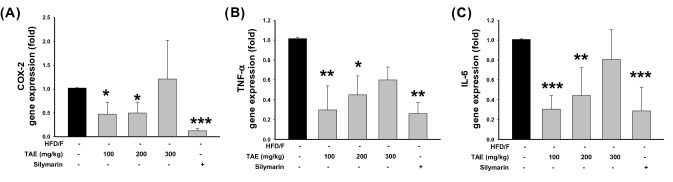
Effect of WS-S on anti-inflammatory cytokines in NAFLD-induced mice. **a** COX-2, **b** TNF-α, and **c** IL-6 relative mRNA levels in the liver. Data are presented as the mean ± SD, n = 8. *p < 0.05, **p < 0.01, and ***p < 0.001 compared to the HFD/F group

Fatty liver disease is closely associated with chronic hepatitis. In experimental animals with fatty liver induced by a HFD, the activity of NFκB in the liver tissue was increased, the gene expression of COX, TNF-α, and interleukin 6 (IL-6) and Kupffer cells and several inflammatory markers were activated (Arab et al., 2019). In patients with steatohepatitis, the level of TNF-α is elevated in the blood and liver tissue, and elevated TNF-α level causes inflammation and insulin resistance (Browning and Horton, 2004). Finally, inhibition of TNF-α improves insulin resistance and histological markers of steatohepatitis (Leyva-Jiménez et al., 2020). Serum IL-6 levels are also increased in insulin resistance and NAFLD with increased hepatitis and liver fibrosis. The expression levels of COX-2, TNF-α, and IL-6 were measured to assess the role of WS-S in inhibiting inflammation. WS-S administration significantly decreased the hepatic mRNA expression levels of COX-2, TNF-α and IL-6 compared with the HFD/F-CON group at concentrations of 100 and 200 mg/kg. (Fig. 4). On the other hand, no significant difference in COX and cytokines was observed in the 300 mg/kg WS-S treated group. Progressive increases in drug dose produce increasing response but only within a relatively narrow range of dose, further increases in dose beyond this range produce little extra effect. WS-S is suggested to have reduced effects on some hepatitis and lipid accumulation improvement at concentrations of 300 mg/kg and above.

### Effects of WS-S on anti-lipogenic activity in NAFLD-induced mice

**Fig. 5:**
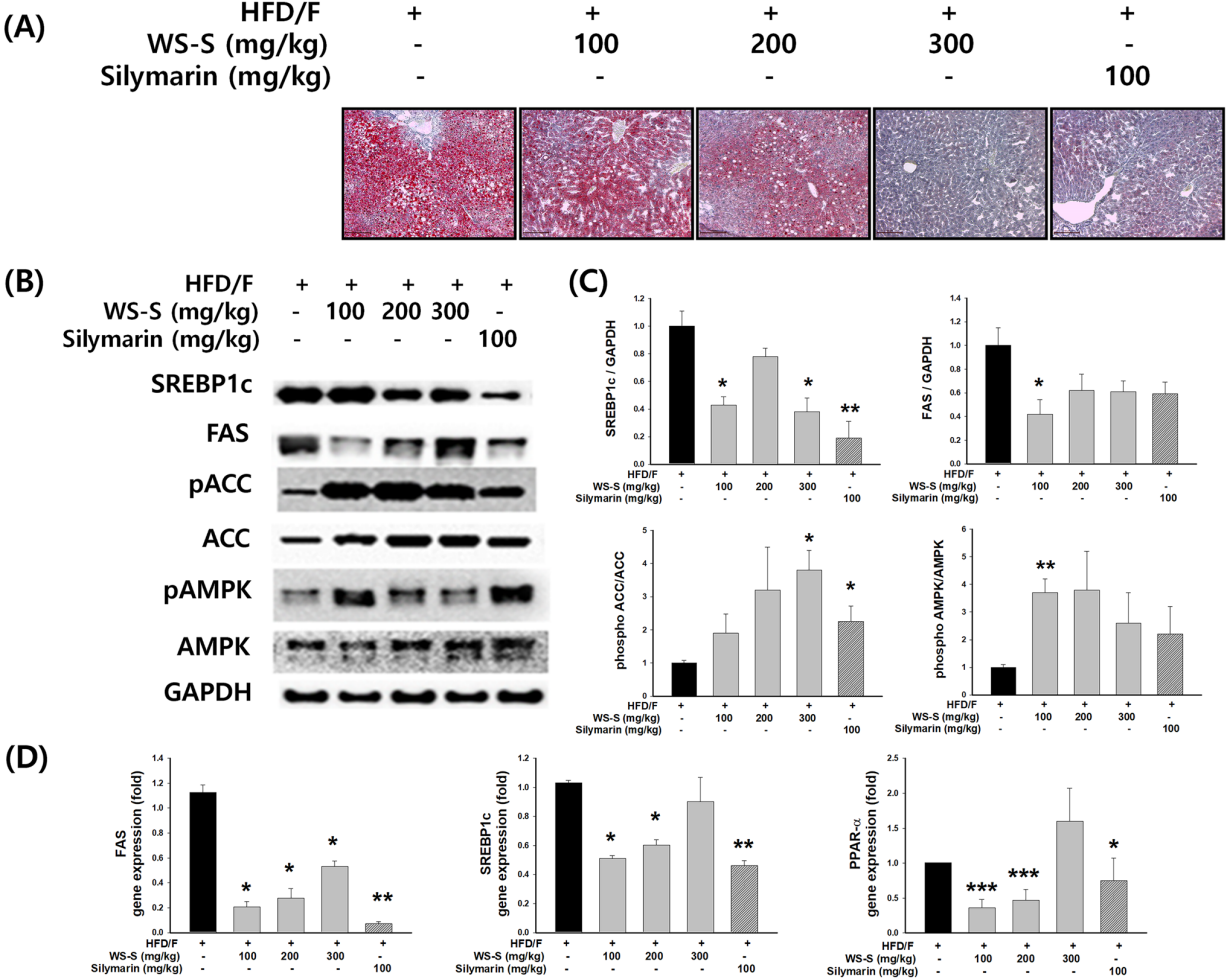
Effect of WS-S on lipid accumulation and lipogenesis in NAFLD-induced mice. **a** Oil red O and hematoxylin staining of the liver. **b** Western blot analysis of SREBP-1c, FAS, phosphoACC, total ACC, phospho AMPK, and total AMPK. **c** Relative protein levels of SREBP-1c, FAS, phospho ACC/ACC ratio, and phospho AMPK/AMPK ratio. GAPDH control verified equal loading of protein. **d** Quantitative real-time PCR analyses of SREBP-1c, FAS, and PPARα were normalized by GAPDH as an internal control. The results of three independent experiments are represented as the mean ± SD. *p < 0.05, **p < 0.01 and ***p < 0.05 compared to the HFD/F group

The effects of WS-S on hepatic lipid accumulation in the liver were detected by Oil Red O staining of histological sections (Fig. 5a). Hepatic intracellular lipid accumulation in the HFD/F-CON group increased, whereas administration of WS-S (100, 200, and 300 mg/kg) significantly ameliorated hepatic intracellular lipid accumulation in the HFD/F-CON group.

Previous other studies (Im et al., 2015; Ziqubu et al., 2020) determined that general *triticum aestivum* sprouts and isoorientin, the main acitve compound of WS-S show lipid accumulation-improving effects via the AMPK pathway. The fatty acid composition of the liver can affect the extent of triglycerides accumulated by altering AMPK activity. AMPK is activated and reduces the expression of SREBP-1c, leading to the inhibition and phosphorylation of ACC and ChREBP (Olzmann and Carvalho, 2019). Therefore, considering the key role of AMPK activation in regulating lipid metabolism, we hypothesized that AMPK plays a vital role in mediating the effects of WS-S against hepatic lipid accumulation.

We evaluated AMPK signaling pathways using western blot analysis and real-time PCR in the hepatic tissue of HFD/F-induced mice to elucidate the molecular mechanisms underlying the anti-lipogenic effects of WS-S. The protein and mRNA expression levels of SREBP1c increased in the HFD/F group and were significantly decreased by WS-S administration. Subsequently, FAS mRNA levels, which are downstream of SREBP-1c, also increased. However, the expression levels of FAS protein and mRNA were significantly lower in the WS-S-treated group than those in the HFD/F group. WS-S inhibition of adipogenesis and changes in the expression of AMPK and ACC, acting downstream of AMPK, were examined, confirming the effect of inhibiting AMPK activity on WS-S induced changes. In addition, the phosphorylation of AMPK and ACC significantly increased (Fig. 5b, c). Wheat sprouts triggered an increase in the phosphorylation of AMPK and its direct substrate, ACC, indicating that WS-S activated AMPK.

Increasing fatty acid β-oxidation may be feasible for reducing hepatic lipid accumulation. PPAR-α is involved in β-oxidation (Kirpich et al., 2012). PPAR-α is an enzyme that transports FFA to the mitochondria for oxidation to generate acetyl-CoA, which then generates ATP through the tricarboxylic acid cycle and electron transport chain. We found that WS-S treatment influenced protein expression.

The results of the present study demonstrate that wheat sprout standardized cultivation (WS-S) decreased hepatic lipid accumulation in vitro and in vivo. WS-S also inhibited the release of pro-inflammatory cytokines, such as COX, TNF‐α, and IL‐6. The inhibition mechanism of hepatic lipid accumulation by WS-S was mainly associated with its suppression of lipogenic genes, including SREBP1c, FAS, and ACC, through the phosphorylation of the AMPK pathway. Therefore, WS-S may be a potential therapeutic agent to treat NAFLD.
